# Behavior of surface contamination by *Salmonella* spp. on broiler carcasses during slaughter stages

**DOI:** 10.1007/s42770-026-02061-0

**Published:** 2026-09-23

**Authors:** Jhennifer Arruda Schmiedt, Leonardo Ereno Tadielo, Emanoelli Aparecida Rodrigues dos Santos, Vinicius Cunha Barcellos, Luiz Gustavo Bach, Victor Hugo Cortez Dias, Monique Ribeiro Tiba Casas, Rafaela de Melo Tavares, Ricardo Seiti Yamatogi, Luís Augusto Nero, Luciano dos Santos Bersot

**Affiliations:** 1 Department of Veterinary Sciences, Federal University of Paraná (UFPR), Rua Pioneiro, 2153, Jardim Dallas, Palotina, PR 85953-128 Brazil; 2 Department of Veterinary Medicine, Santa Catarina State University (UDESC), Lages, Santa Catarina 88520-000 Brazil; 3 School of Veterinary Medicine and Animal Science, São Paulo State University (UNESP), Distrito de Rubião Jr, Botucatu Campus, Botucatu, SNSP 18618-970 Brazil; 4 Adolfo Lutz Institute, Bacteriology Center, Avenida Doutor Arnaldo, 351, 9th Floor, Sumaré, São Paulo, SP 01255-000 Brazil; 5 Department of Veterinary Medicine, Federal University of Viçosa, University Campus, Viçosa, Minas Gerais 36570-000 Brazil

**Keywords:** Slaughterhouse, Poultry chain, PFGE, Pathogen, Salmonellosis, Heidelberg

## Abstract

*Salmonella* spp. is a globally relevant pathogen frequently associated with foodborne infections and poultry products. This study aimed to evaluate the occurrence and quantification of *Salmonella* spp. during different stages of the broiler slaughter process and to assess the association of processing stages with reductions in contamination levels. The study was conducted in a federally inspected broiler slaughterhouse in Paraná, Brazil. A total of 2,100 analyses were performed on chicken carcasses collected at seven points along the slaughter line over 20 weeks. *Salmonella* detection followed the ISO 6579:2017 methodology, while quantification was performed using the miniaturized most probable number (mMPN) method with molecular confirmation by the invA gene. Subsequently, 211 isolates were serotyped, and 97 *Salmonella* Heidelberg isolates were analyzed by pulsed-field gel electrophoresis (PFGE). The highest occurrence of *Salmonella* spp. was observed at the pre-scalding stage (*N* = 236/78.66%). Significant reductions in *Salmonella* counts were observed after the scalding stage, after the post-evisceration critical control point for removal of visibly contaminated carcasses (CCP 1B), and after final washing before chilling (*P* < 0.05). Five serovars were identified, with *Salmonella* Heidelberg predominating (*N* = 193/91.5%). PFGE analysis revealed high genetic similarity (90%) among isolates, with highly similar profiles identified at different slaughter stages and sampling weeks. These findings demonstrate that *Salmonella* spp. was detected throughout the slaughter process, although reductions in contamination levels were observed at specific processing stages. In addition, the detection of highly similar isolates at different sampling points and weeks contributes to the understanding of Salmonella contamination dynamics in poultry slaughterhouses.

## Introduction

The Brazilian agribusiness sector plays an important role in the global food production scenario, with notable emphasis on the poultry chain. In 2024, chicken meat production reached 14.972 million tonnes, resulting from the slaughter of 5.449 billion chickens [1]. This high level of chicken meat production is supported by a consolidated industrial production system based on biosecurity, control, and prevention of pathogenic agents. In this context, the use and implementation of control plans on an industrial scale aim to reduce or eliminate chemical, physical, and biological hazards during production [6, 27].

Biological hazards are responsible for many preventive and control actions, particularly due to the presence of microorganisms of public health importance, such as *Salmonella* spp. [19]. Data recently compiled by the Food Safety and Inspection Service indicate that *Salmonella* spp. is one of the main causes of diseases resulting from the consumption of contaminated food [61]. According to data from the National Health Surveillance Agency of the Ministry of Health, *Salmonella* is the third most frequently reported pathogen associated with foodborne outbreaks in Brazil [8].

Contamination of carcasses and industrial contact surfaces by *Salmonella* spp. may occur at different stages of the poultry slaughter process [13, 51]. Some stages are considered critical points for bacterial contamination, particularly due to the tendency for microorganism dissemination among carcasses. Among these stages, scalding, defeathering, evisceration, pre-chilling, and carcass chilling stand out [17, 38, 48, 49, 57, 70].

Scalding, whose primary technological purpose is to facilitate feather removal, may also contribute to the removal of dirt and the reduction of microbial load; however, it can also become a source of *Salmonella* contamination due to the presence of organic matter in the scalding water [2, 15, 25], as may defeathering, which, when performed with poorly sanitized equipment, can intensify this dissemination [3, 38, 49, 57]. During evisceration, rupture of the viscera represents a high risk of contamination along the slaughter line [14, 49, 11]. Finally, carcass chilling in tanks with continuous water flow may favor the spread of the pathogen among products, given the direct contact between carcasses within the system [33, 55, 66, 68]. One of the difficulties in controlling Salmonella in the industry is related to the tolerance of these microorganisms, which, even after sanitation, may remain on equipment, contributing to cross-contamination [43, 64]. 

Thus, several studies have sought to evaluate and propose techniques and methods to reduce the risks of *Salmonella* contamination [42]. In an applied context, these studies should support the updating and improvement of industry control plans, which should include routine assessments of the entire process, identifying areas of higher risk and proposing variables for their control [6, 7, 10, 12, 41]. Among these variables, the quantification of *Salmonella* spp. is not commonly performed within the poultry slaughter process, with few references available [4, 40, 53].

Although numerous studies have reported the prevalence of *Salmonella *on broiler carcasses at critical processing stages [5, 25, 34, 48], few have combined quantitative enumeration with longitudinal monitoring throughout the entire commercial slaughter process. Furthermore, studies integrating contamination quantification, evaluation of operational processing variables, serovar distribution, and genetic relatedness under routine industrial conditions remain scarce. Consequently, there is limited understanding of how *Salmonella *contamination levels fluctuate throughout slaughter, which processing steps are most effective in reducing microbial loads, and whether closely related strains persist and disseminate over time within the same processing plant.

To address these knowledge gaps, the present study conducted a 20-week longitudinal monitoring of a commercial Brazilian broiler slaughterhouse, integrating qualitative detection, quantitative enumeration, assessment of operational processing variables, serovar identification, and PFGE analysis of the predominant serovar. This integrated approach provides a more comprehensive understanding of *Salmonella* contamination dynamics throughout commercial processing and generates epidemiological evidence that may support improvements in microbiological control strategies across the poultry production chain.

The objective of this study was to evaluate the occurrence and quantification of *Salmonella* spp. throughout the different stages of the broiler slaughter process and to assess the association of processing stages with reductions in contamination levels. In addition, operational variables such as slaughter shift, carcass weight, fasting time, age, and shackle line speed were analyzed, as well as their influence on the microbial load throughout the slaughter flow. Finally, the study aimed to characterize the prevalent serovars and the genetic similarity among the isolates recovered during the process.

## Materials and methods

### Sampling and data collection

This study was conducted between August 2020 and March 2021 in a large-scale slaughterhouse with official inspection service and authorized for export, located in the state of Paraná, Brazil. The operational slaughter capacity was 400,000 birds per day, distributed across two work shifts. To evaluate the occurrence of *Salmonella* spp., 15 chicken carcasses were sampled weekly over a 20-week period. Following the same carcasses throughout the slaughter process ensured a standardized assessment of microbiological changes associated with each technological operation, whereas comparisons among processing stages were performed using the pooled data obtained over the 20-week monitoring period. At each weekly sampling, 15 carcasses were randomly selected and individually identified before slaughter. The same carcasses were subsequently followed throughout the slaughter process and sampled at each of the seven processing stages, resulting in 15 samples per sampling point per week and a total of 105 samples collected each week (15 carcasses × 7 sampling points). The seven sampling points were: pre-scalding (A); post-scalding (B); post-defeathering (C); post-evisceration before the critical control point (CCP 1B) (D); post-evisceration after CCP 1B (E); post-final washing before the chiller (F); and post-chilling (G). CCP 1B corresponded to the critical control point established by the slaughterhouse for visual inspection and removal of carcasses showing visible fecal, biliary, or gastrointestinal contamination after evisceration, according to the plant’s HACCP program. The chilling stage consisted of immersion chilling under continuous water flow at temperatures maintained according to Brazilian industrial standards. No antimicrobial chemical compounds were used during the chilling process. At each point, the carcasses were marked, evaluated, and subsequently returned to the shackle line, totaling 300 carcasses analyzed per point and, overall, 2,100 microbiological analyses were performed. Following the same carcasses throughout the slaughter process ensured a standardized assessment of microbiological changes associated with each technological operation, whereas comparisons among processing stages were performed using the pooled data obtained over the 20-week monitoring period.

At each sampling, the work shift, shackle line speed (carcasses/hour), fasting time of the flock (hours), and age of the birds (days) were recorded. The individual weight (grams) of each carcass was obtained using a semi-analytical balance with a precision of 0.001 g. The slaughter line speed varied according to routine industrial operational adjustments, including flock size, equipment maintenance, and production demand, ranging from 7,000 to 10,800 birds/hour. For statistical purposes, processing speeds were categorized into two operational groups based on the plant's standard commercial processing conditions: ≤ 10,500 and > 10,500 birds/hour. Sampling was scheduled on days when no sanitary slaughter (defined as batches positive for *Salmonella* in official tests carried out on the farm) was performed, to ensure uniformity of the evaluations.

For sample collection, the surface rinse method was used, in which carcasses were subjected to a surface rinsing technique with 400 mL of 1% buffered peptone water. Prior to rinsing, carcasses were weighed and the weights recorded in spreadsheets for contamination calculation. Samples were collected during the first shift (seven samples) and the second shift (eight samples), totaling 15 carcass samples per point each week. The samples were placed in insulated containers, kept under refrigeration, and transported to the Laboratory of Inspection and Quality Control of Food and Water (LACOMA) at the Federal University of Paraná for microbiological analyses.

### Quantification and detection of *Salmonella spp*.

The quantification of *Salmonella* spp. was performed using the miniaturized most probable number (mMPN) method, according to the methodology described in ISO/TS 6579-2:2012, with modifications [29]. After homogenization of 400 mL of buffered peptone water (BPW, Difco™) for each carcass (item 2.1.1), three 2.5 mL aliquots were distributed into three wells of a 24-well cell culture microplate. Subsequently, 500 μL from each initial well was transferred to subsequent wells containing 2 mL of BPW, obtaining the 10⁻^2^ dilution. The procedure was repeated serially for two additional steps until reaching the 10⁻^4^ dilution.

The microplates were incubated at 36 ± 1 °C for 18 ± 2 h. After incubation, 20 μL from each well was transferred to plates containing semisolid Rappaport–Vassiliadis medium (MSRV, Difco™) and incubated at 41.5 ± 1 °C for 24–48 h. Wells that showed a growth halo during this period were considered presumptively positive for *Salmonella* spp. and were confirmed by streaking onto xylose lysine deoxycholate agar (XLD, Difco™), followed by incubation at 36 ± 1 °C for 24 ± 3 h.

From plates showing characteristic growth of *Salmonella* spp., three characteristic colonies were selected and subjected to biochemical and serological confirmation by slide agglutination with polyvalent somatic O antiserum. When results were characteristic of the genus *Salmonella*, molecular confirmation was performed by detection of the *invA* gene using the polymerase chain reaction (PCR) technique [60]. If any of the three tested colonies showed results compatible with *Salmonella* spp., the well from which that colony was obtained was considered positive. The numerical results of the mMPN technique were obtained using the MPN calculation program, version 6, and expressed as MPN/g of the sample.

For the evaluation of *Salmonella* spp. occurrence, the methodology described in ISO 6579-1:2017 was used [28]. Accordingly, the remaining volume of the samples intended for quantification was incubated at 36 ± 1 °C for 18 h. Subsequently, aliquots of 1 mL and 0.1 mL of the pre-enriched sample were transferred, respectively, to tubes containing 10 mL of Müller–Kauffmann tetrathionate broth supplemented with novobiocin (Merck) and Rappaport–Vassiliadis soya peptone broth (Oxoid), and incubated at 36 ± 1 °C and 42.5 °C for 24 h. After selective enrichment, samples were streaked onto xylose lysine deoxycholate agar (XLD, BD Difco™) and bismuth sulfite agar (BS, BD Difco™), followed by incubation at 36 ± 1 °C for 24 h. Three characteristic colonies from each medium were selected for biochemical confirmation. Isolates with a compatible profile were subjected to slide agglutination with polyvalent antiserum, and the results were expressed as the presence or absence of *Salmonella* spp. in 25 mL of sample.

### Serotyping and Pulsed-Field Gel Electrophoresis (PFGE)

A total of 211 *Salmonella* isolates were selected for serotyping using a stratified proportional sampling strategy designed to ensure representative coverage of the 20 sampling weeks and the seven slaughter processing stages. Between 10 and 11 isolates were selected from each sampling week and distributed proportionally among the sampling points based on the occurrence of positive samples. Thus, whenever possible, at least one isolate from each processing stage was included per week, ensuring representative coverage of the temporal distribution and processing stages throughout the study. Serotyping was performed using somatic (O) and flagellar (H, phases 1 and 2) antisera according to the White–Kauffmann–Le Minor scheme [24, 30]. The analyses were conducted at the Center for Enteric Diseases and Infections by Special Pathogens (NDEI) of the Bacteriology Center of the Adolfo Lutz Institute (IAL/SP).

To elucidate the circulation of *Salmonella* across slaughter stages and assess its temporal distribution, the most prevalent serotypes from the study were selected. Of the 211 isolates subjected to serotyping, 193 were identified as S. Heidelberg (91.5%); for PFGE analysis, 97 S. Heidelberg isolates were selected using a stratified sampling approach. This selection ensured a comprehensive macro-restriction profile analysis and representative coverage across the 20 weeks of sampling and all slaughter processing stages evaluated, prioritizing the inclusion of isolates from different weeks and processing points whenever available.

PFGE was performed according to the PulseNet protocol described by Ribot et al. [50], using **XbaI** (Fermentas, Life Sciences) as the restriction enzyme. Electrophoresis was carried out using a CHEF-DR® III system (Bio-Rad®). After electrophoresis, the gels were stained with GelRed (Biotium), and banding patterns were visualized under UV transillumination. The images were analyzed using BioNumerics® software version 7.1 (Applied Maths, Sint-Martens-Latem, Belgium), with a position tolerance and optimization of 1.5%. Similarities among profiles were calculated using the Dice coefficient, and the dendrogram was generated using the UPGMA clustering method (unweighted pair group method with arithmetic averages).

### Statistical analysis

Normality of the *Salmonella* spp. occurrence data was assessed using the Shapiro–Wilk and D’Agostino–Pearson tests. As the assumptions of normality were not met, nonparametric tests were used. Differences among sampling points and weeks were analyzed using the Kruskal–Wallis test, followed by Dunn’s post hoc test when significant differences were identified (*P* < 0.05).

For the quantification of *Salmonella* spp., log MPN/g values and environmental variables were subjected to descriptive statistical analysis by slaughter stage. Analysis of variance was used to compare means, and differences were determined by Tukey’s test (*P* < 0.05). The K-means procedure was used to determine weight groups. All analyses were performed using SPSS software, version 19. The statistical analyzes were selected according to the study objective of comparing the average microbiological performance among the different slaughter stages throughout the monitoring period, with each sampling week considered an independent replication of the production process.

## Results and discussion

**Fig. 1:**
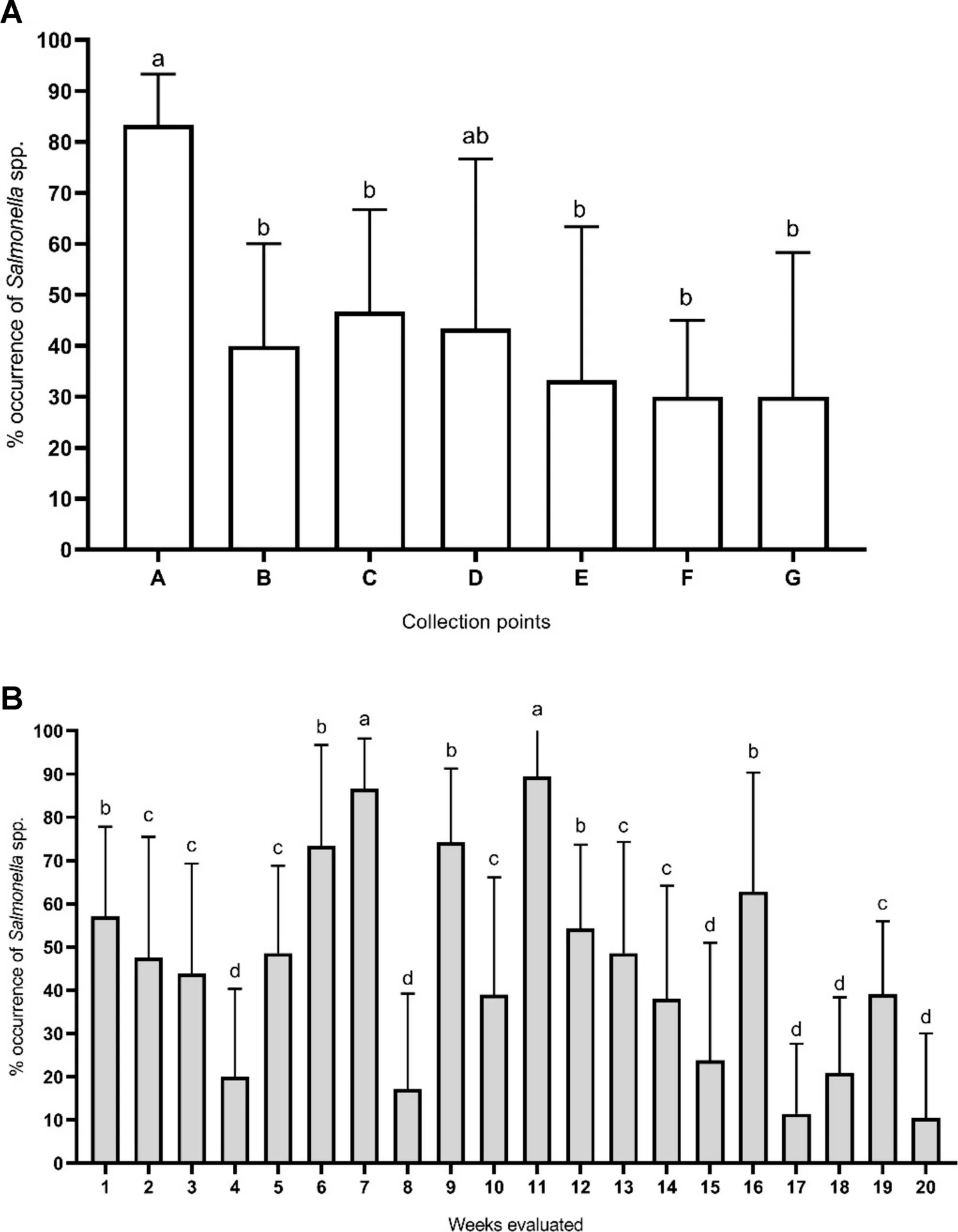
Occurrence (%) of *Salmonella* spp. at different stages of the broiler slaughter process (**A**) and according to the evaluated sampling weeks (**B**). A total of 300 carcasses were analyzed per sampling point, totaling 2,100 microbiological analyses overall. Bars represent median values, and error bars indicate the interquartile range. Different letters indicate significant differences according to the Kruskal–Wallis test followed by Dunn’s multiple comparisons test (*P* < 0.05). A, pre-scalding; B, post-scalding; C, post-defeathering; D, post-evisceration before the critical control point CCP 1B; E, post-evisceration after CCP 1B; F, post-final washing before the chiller; G, post-chilling

The overall occurrence of *Salmonella* was 45.3% (*n* = 952) and differed significantly among the evaluated sampling points (*P* < 0.0001). The pre-scalding point showed a significantly higher occurrence (78.66%) compared to the post-scalding (42.66%), post-defeathering (43.01%), post-evisceration after CCP 1B (38.01%), post-final washing before the chiller (28.99%), and post-chilling (36.66%) points (*P* < 0.05), while it did not differ from the post-evisceration before the critical control point CCP 1B (49.33%) Fig. 1A. The post-scalding, post-defeathering, post-evisceration after CCP 1B, post-final washing before the chiller, and post-chilling points did not show statistically significant differences among themselves, characterizing a homogeneous group regarding pathogen occurrence Fig. 1A. According to the sampling weeks, considering the sampling points as replicates, the mean occurrence of *Salmonella* differed statistically (*P* < 0.0001). Weeks 7 (86.66%) and 11 (89.53%) showed the highest occurrences, whereas weeks 17 (11.43%) and 20 (10.47%) showed the lowest occurrences (*P* < 0.05) Fig. 1B. The remaining weeks presented intermediate values (ranging from 20.96% to 73.33%), with no statistical differences among them Fig. 1B.

There was a significant reduction (*P* < 0.05) of 0.97 log MPN/g in the quantification of *Salmonella* spp. when comparing the logarithmic mean at entry (point A, pre-scalding) and exit (point G, post-chilling), with values of 1.30 and 0.33 log MPN/g, respectively. These data indicate that the technological slaughter process is effective in reducing counts, but not in eliminating the hazard.

**Fig. 2:**
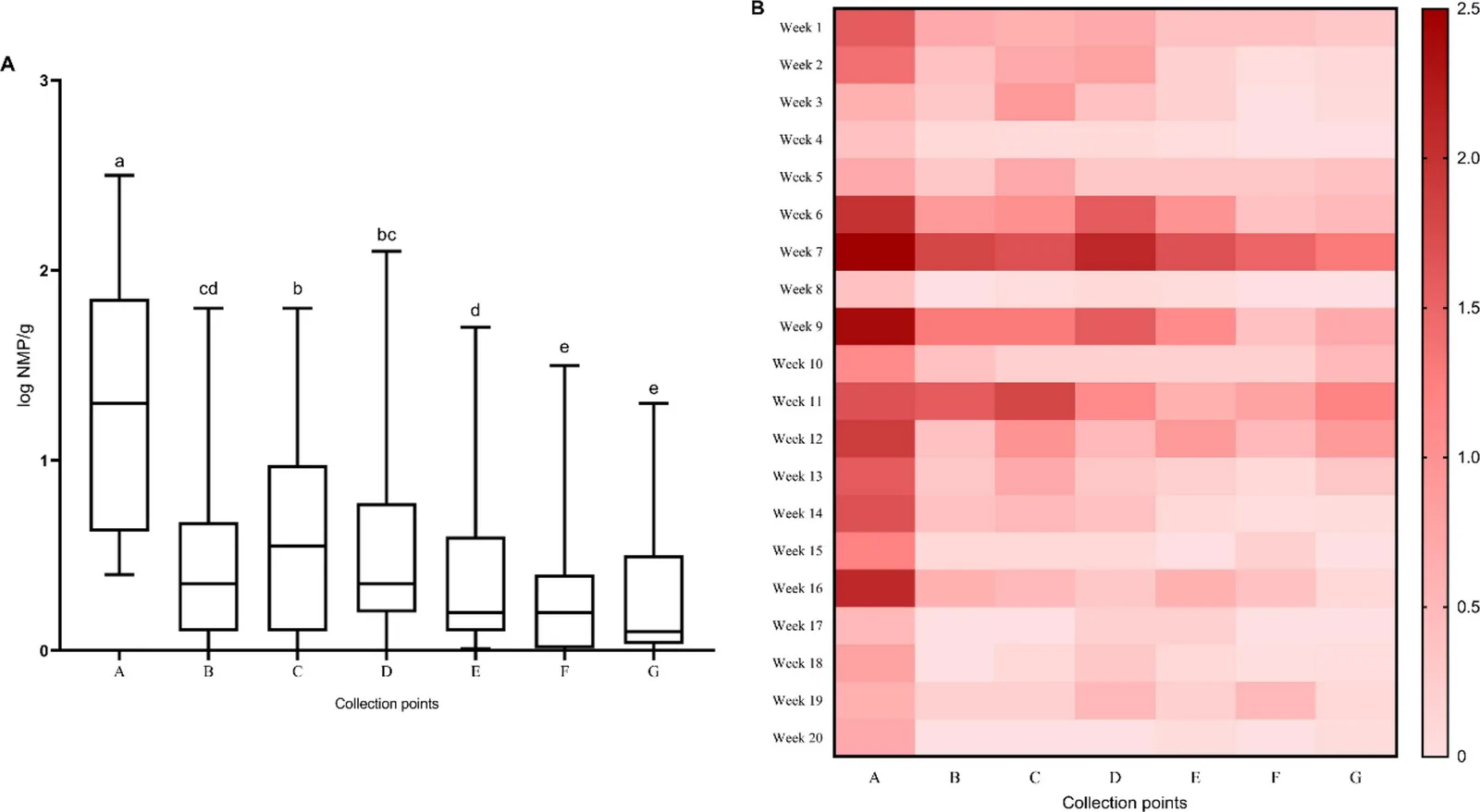
Quantitative distribution and temporal dynamics of *Salmonella* spp. contamination along the broiler slaughter process. (**A**) Boxplots representing the distribution of *Salmonella* spp. counts expressed as log MPN/g at each collection point: pre-scalding (A), post-scalding (B), post-defeathering (C), post-evisceration before CCP 1B (D), post-CCP 1B (E), post-final washing (F), and post-chilling (G). Boxes indicate the interquartile range, horizontal lines represent medians, and whiskers indicate minimum and maximum values. Different lowercase letters indicate statistically significant differences among collection points (*P* < 0.05). (**B**) Heatmap showing the weekly mean *Salmonella* spp. counts (log MPN/g) at each collection point over 20 consecutive sampling weeks. Color intensity represents the magnitude of contamination, with green indicating lower counts and red indicating higher counts

Figure 2 shows high standard deviation values across all slaughter stages, indicating substantial variability in *Salmonella* counts among sampling days and carcasses. This variability may reflect the heterogeneous distribution of *Salmonella* contamination during poultry processing, as well as the longitudinal sampling design adopted in the present study, which included weekly collections and 15 carcasses evaluated per sampling point. In addition, the study did not include environmental or farm-level sampling, limiting the ability to determine the specific sources contributing to this variation. Significant reductions occurred between the pre- and post-scalding points (A–B), post-evisceration before and after CCP 1B (D–E), and post-evisceration after CCP 1B and post-final washing (E–F), demonstrating that the scalding stage, CCP 1B, and final washing were associated with reductions in *Salmonella* counts under the evaluated processing conditions (*P* < 0.05) Fig. 2.

The initial point, pre-scalding, showed the highest level of contamination, which was already expected, as this contamination reflects the surface contamination of live birds that carry this material from the rearing environment, including the presence of feces and excess organic matter on their feathers. These results were similar to those reported in studies by Rivera-Pérez et al., Yamatogi et al., Boubendir et al. [5, 36, 49, 67], and Marín et al., conducted in slaughterhouses located in Costa Rica, Brazil, Canada, and Spain, respectively.

The significant reduction in the overall mean *Salmonella* spp. count from the pre-scalding point to the subsequent points indicates that the scalding process contributed to the reduction of *Salmonella* on chicken carcasses Fig. 2. Some studies have shown that scalding water temperature is related to *Salmonella* inactivation, with temperatures between 55 °C and 60 °C considered effective in reducing the microbial load of this pathogen [56, 69]. The scalding water temperature in the evaluated slaughterhouse was maintained, on average, at 60 °C, which may have contributed to the reduction in *Salmonella* counts observed after the scalding stage, with a reduction of 0.81 log MPN/g between the pre- and post-scalding points (A–B).

The evisceration and pre-chilling stages did not maintain reduced contamination levels. There was a significant increase in mean contamination between the post-scalding and post-defeathering points (0.11 log MPN/g), suggesting that the defeathering stage contributed to this increase. Some slaughter stages, such as defeathering, may favor increased contamination and dissemination of *Salmonella*, especially when failures occur during the process or when inadequate sanitation of the defeathering equipment is observed, reinforcing that this stage is among those most frequently implicated in carcass contamination [2, 3, 38, 70].

The final washing step (point E–F) promoted a significant reduction of 0.12 log MPN/g in the overall mean *Salmonella* spp. count. Other studies corroborate these findings and reported that carcass washing after the evisceration process resulted in satisfactory reductions of *Salmonella* spp. and indicator microorganisms [23, 35, 37, 62]. Despite the significant effect of final washing applied to the carcasses, an increase in *Salmonella* contamination was observed after the chilling process (point G, post-chilling). In a study using *Escherichia coli* as the indicator microorganism, Da Silva et al. [18] also observed a reduction in contamination after final washing,however, following passage through the chilling system, an increase in contamination occurred, interfering with the microbiological reduction achieved during the final washing step.

Similar results were obtained by Smith, Cason, and Berrang [58], whose study evaluated the effects of fecal contamination and cross-contamination on the numbers of coliforms, *Escherichia coli*, *Campylobacter*, and *Salmonella* in immersion-chilled broiler carcasses, demonstrating an increase in cross-contamination after chilling. These findings indicate that the chilling process may represent a critical point for the proliferation of pathogens such as *Salmonella*. In addition, Wang et al. [62] evaluated the bacterial diversity present during broiler slaughter and processing and demonstrated an increase in bacterial populations on carcasses after the chilling stage.

Regarding the 20 sampling weeks, during which 15 carcasses were collected per point on each sampling date, it was possible to observe that the four highest values at point G (post-chilling) were obtained in weeks 7, 9, 11, and 12, with mean detected counts of 1.3, 0.7, 1.2, and 0.9 log MPN of *Salmonella*/g, respectively Fig. 2. In these sampling weeks, the initial point (point A, pre-scalding) showed higher *Salmonella* contamination. These results suggest that final product contamination reflects the incoming contamination (point A, pre-scalding). In these sampling weeks, surface contamination of chicken carcasses also showed high counts at the post-defeathering (point C) and post-evisceration before CCP 1B (point D) points, suggesting that failures may have occurred during the defeathering and evisceration stages, contributing to increased carcass contamination at subsequent points.

However, contamination at the pre-scalding point during weeks 1, 2, 6, 13, 14, and 16 was statistically similar to that observed in the previously mentioned weeks; however, the high contamination pattern was not maintained throughout slaughter, as low counts were observed at the post-chilling point. This indicates that high initial contamination does not necessarily result in a more contaminated final product, as it may reflect the effectiveness of the slaughter stages which, if properly executed without technological failures or cross-contamination, result in low final contamination.

By comparing the occurrence and quantification results of *Salmonella* spp., it is possible to confirm the effectiveness of the scalding process. In contrast, stages such as defeathering and evisceration showed increases in *Salmonella* counts without proportional increases in occurrence, highlighting their role as critical points for cross-contamination that would not be fully captured by qualitative analyses alone.

**Fig. 3:**
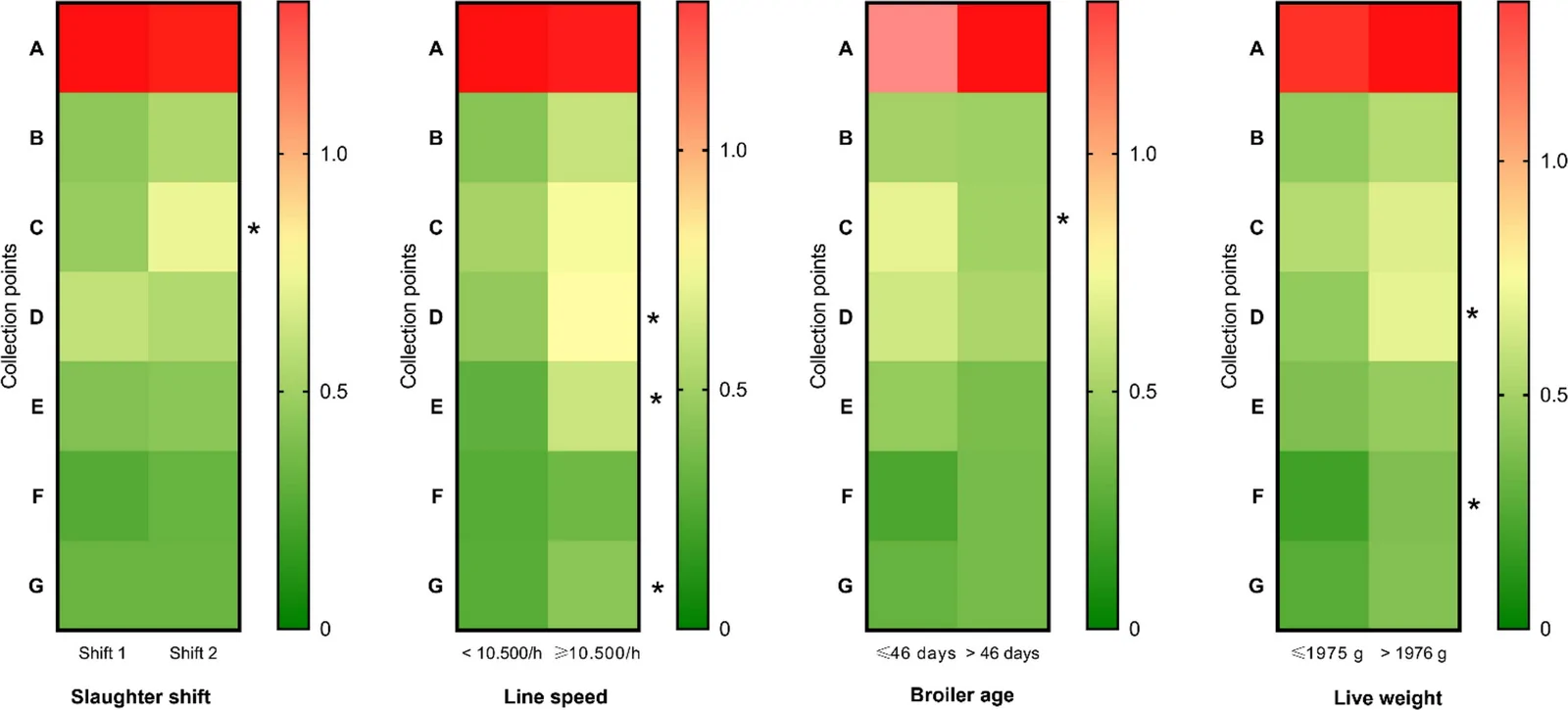
Evaluation of the effect of slaughter shift, speed, age, and bird weight on *Salmonella* spp. counts (log MPN/g) at each of the different slaughter stages evaluated. * indicates statistically significant differences within the same slaughter stage (*P* < 0.05). A – pre-scalding; B – post-scalding; C – post-defeathering; D – post-evisceration before CCP 1B; E – post-CCP 1B; F – post-final washing; G – post-chilling. *N* = number of samples; SD = standard deviation

Other factors, such as shackle line speed, slaughter shift, fasting time, age, and carcass weight, may contribute to the increase and dissemination of *Salmonella* in the industry [26]. Regarding environmental variables such as slaughter shift (1st and 2nd), no effect on *Salmonella* contamination was observed Fig. 3, except at the post-defeathering stage (point C), where *Salmonella* counts obtained during the 2nd shift were higher than those from the 1 st shift (*P* < 0.05).

Figure 3 shows that higher slaughter speed was associated with higher *Salmonella* counts (*P* < 0.05) at the post-evisceration before CCP 1B (point D), post-CCP 1B (point E), and post-chilling (point G) stages, demonstrating that both the evisceration stage and the CCP 1B monitoring operation were affected by processing speed, as maintaining quality becomes more difficult under these conditions. Thus, higher slaughter speed was associated with higher *Salmonella* counts per gram of carcass at these stages. It can be noted that higher slaughter speed affects verification of the critical control point CCP 1B, which, as a visual inspection step, may hinder the detection of carcass contamination and consequently lead to increased contamination after this stage.

Although feed withdrawal is widely recognized as an important management practice to reduce gastrointestinal content and minimize carcass contamination during evisceration, no significant association between fasting time and *Salmonella* counts was observed in the present study. This result may be related to the limited variation in fasting times among the evaluated flocks or to the multifactorial nature of contamination during processing, which also involves operational and equipment-related factors. The influence of age Fig. 3 showed higher contamination (*P* < 0.05) in carcasses obtained from birds slaughtered at ≤ 46 days of age, only at stage C (post-defeathering).

Bird weight Fig. 3 was significantly associated with contamination observed at the post-evisceration before CCP 1B (point D) stage and at the final washing stage (point F); in this case, higher bird weight was associated with higher contamination during evisceration. Therefore, carcass uniformity is essential to ensure proper machine operation and to reduce carcass contamination, especially fecal–biliary contamination [59].

Among the *Salmonella* isolates that were serotyped (*n* = 211), five serovars with higher prevalence were identified, with *S. *Heidelberg being the most prevalent (91.5%), followed by *S. *Infantis (5.7%), *S. *Minnesota (1.4%), *S. *Coeln (0.9%), and *S. *Saintpaul (0.5%). Regarding the sampling points, at the pre-scalding point (point A), at least one isolate of each serovar was detected. At the post-evisceration before CCP 1B (point D), post-CCP 1B (point E), and post-final washing (point F) points, three different serovars were identified. However, only *S.* Heidelberg was detected at all evaluated sampling points, suggesting that this serovar was consistently present throughout different stages of processing during the study period.

At the pre-scalding stage, all five major *Salmonella* serovars were identified, suggesting that higher enteric contamination originating from the farm environment exhibits such diversity and serves as an important source of contamination throughout the slaughter process. The association between *S. *Heidelberg and *S. *Infantis was the most common, except at the post-defeathering point, where only *S. *Heidelberg was detected. Other studies have reported similar findings, with a high prevalence of *S. *Heidelberg in the Brazilian poultry production chain, especially in the southern region of Brazil, including the states of Rio Grande do Sul and Paraná [16, 21, 22, 44, 46, 47, 52, 54 ]. In 2023, official control carried out in poultry slaughter establishments in Brazil indicated the presence of *Salmonella* spp. in 14.8% of the analyzed chicken samples. Among the identified serovars, *S. *Minnesota was the most prevalent, accounting for 65.76% of cases, followed by *S. *Heidelberg, with 16.10% of occurrences [9].

The high prevalence of S. Heidelberg is also a warning sign, as this serovar is associated with human disease and has been detected in products of animal origin [20, 32, 45]. In addition, the presence of this serovar at all stages of the present study may reflect characteristics that favor its occurrence under poultry production and processing conditions, including tolerance to stress conditions that may influence bacterial survival in the processing environment [31, 39, 63, 65].

**Fig. 4:**
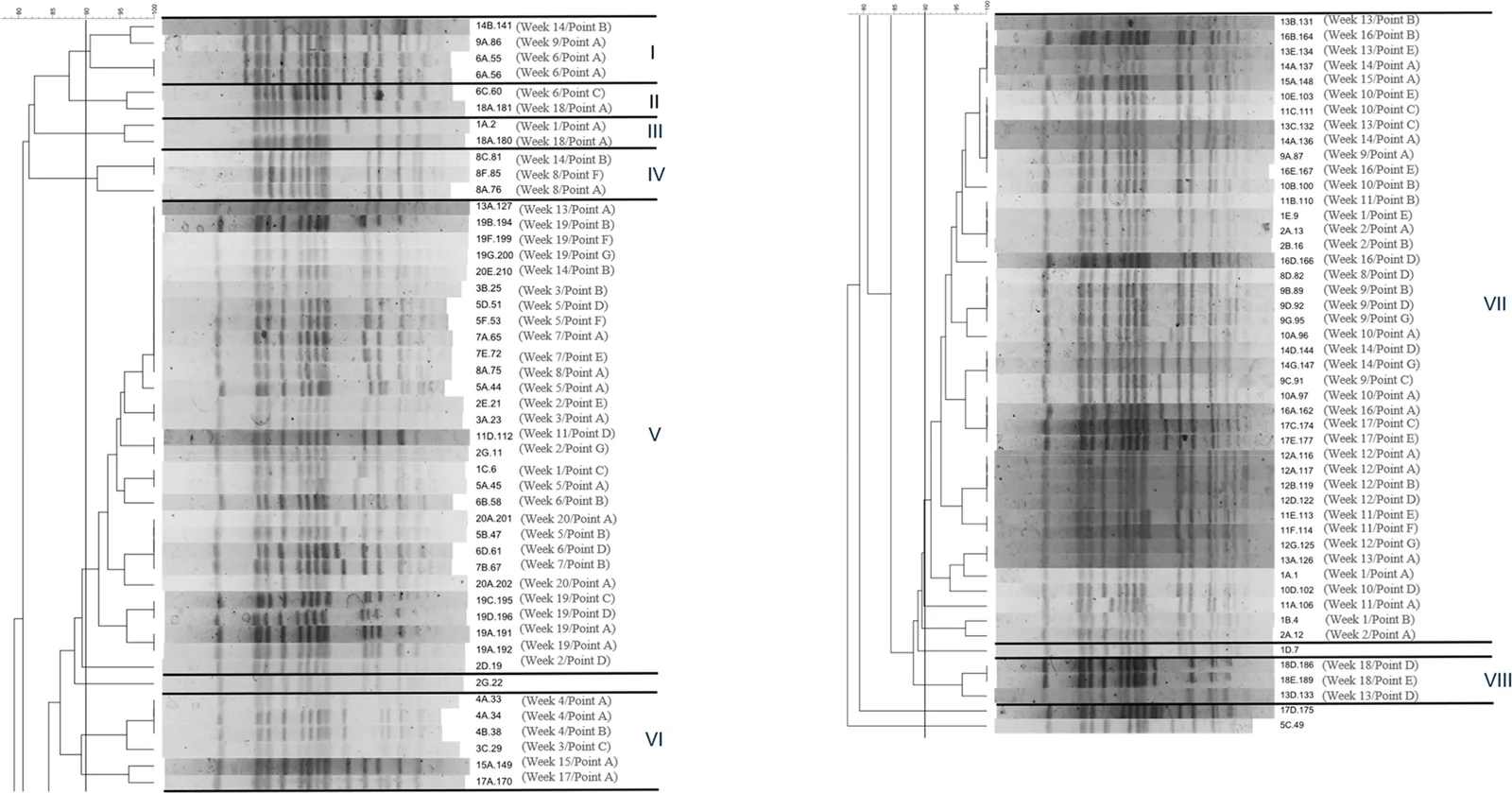
Dendrogram of *Salmonella Heidelberg* isolates (*n* = 97) obtained at different processing stages in a poultry slaughterhouse, using a 90% similarity cutoff. Sampling weeks: 1 to 20; Points: A – pre-scalding; B – post-scalding; C – post-defeathering; D – post-evisceration before CCP 1B; E – post-CCP 1B; F – post-final washing; G – post-chilling. Legend: The first number refers to the week the sample was collected, and the letter to the respective collection point

Due to the high circulation of *S. Heidelberg* at all stages evaluated in the study, as it proved to be a serovar that reflects the *Salmonella* contamination profile throughout the broiler carcass processing flow, a macrorestriction profile analysis was performed on 97 isolates. As a result, eight distinct clusters (I to VIII) were identified based on 90% similarity, as shown in Fig. 4.

Isolates from different weeks and processing stages were identified in all clusters, demonstrating that *Salmonella* isolates originating from distinct points share high genetic similarity, which suggests the viability and dissemination of the pathogen along the slaughter line. In addition, the presence of isolates from different weeks grouped within the same pulsotype reinforces the hypothesis that the same genetic profile persistently circulated in the slaughterhouse over an extended period.

Analysis of clusters V and VII revealed the repeated detection of highly similar isolates throughout the evaluation period, evidenced by the high genetic similarity among *Salmonella* isolates obtained from different sampling weeks and slaughter stages. The presence of isolates originating from different points along the processing line grouped within the same cluster may suggest possible cross-contamination events during slaughter. In cluster V, specifically, isolates obtained in week 19, from point A (pre-scalding) to point G (post-chilling), exhibited highly similar genetic profiles, indicating the detection of closely related *Salmonella* isolates throughout the slaughter process Fig. 4.

Another relevant finding was the detection of isolates with high genetic similarity at critical control points of the process, such as scalding (B), CCP 1B (E), final washing (F), and chilling (G). Although the scalding process promoted a significant reduction in the quantity of *Salmonella* spp., PFGE analysis revealed the detection of highly similar isolates at subsequent stages of processing. These findings indicate that highly similar isolates were detected before and after the scalding stage. In cluster V, highly similar genetic profiles were identified among isolates collected before and after scalding (3A–3B; 5A–5B; 7A–7B; 19A–19B), demonstrating the occurrence of closely related isolates at different stages of the slaughter process.

Considering that *Salmonella* Heidelberg was the serovar most frequently detected in the present study, and that the evaluated broilers originated from different farms, mostly belonging to the same integrated company, the detection of highly similar isolates across different sampling weeks may reflect the widespread occurrence of this serovar within the evaluated production system. This observation is consistent with the repeated detection of isolates with highly similar genetic profiles during the study period, as demonstrated by the high genetic similarity observed among samples collected in different weeks.

A limitation of the present study is that only isolates obtained from broiler carcasses were evaluated, without the inclusion of samples from supplying farms or from the slaughterhouse environment. Therefore, although highly similar PFGE profiles were detected over the monitoring period, it was not possible to determine whether these strains were repeatedly introduced from farms, persisted within the processing environment, or were disseminated through cross-contamination and recirculation events during slaughter. Furthermore, although PFGE remains a standardized and widely accepted method for assessing the genetic relatedness of *Salmonella* isolates, it has lower discriminatory power than whole-genome sequencing (WGS), which is currently considered the reference approach for molecular epidemiological investigations. Future studies should integrate isolates from farms, equipment and contact surfaces, processing water, and other environmental sites within the slaughterhouse and combine genome-based approaches such as WGS to improve source attribution, characterize transmission pathways with greater resolution, and provide a broader epidemiological understanding of *Salmonella* transmission throughout the poultry production chain.

## Conclusion

The results of this study demonstrate that *Salmonella* spp. was detected at all stages of the broiler slaughter process, although its microbiological load varied throughout processing. The reduction in *Salmonella* counts observed between the entry and exit points of the process indicates that the scalding stage, CCP 1B, and post-final washing were associated with reductions in *Salmonella* contamination during processing; however, the pathogen was not completely eliminated. A similar pattern was observed for the overall occurrence of *Salmonella*. In contrast, the defeathering stage showed an increase in *Salmonella* contamination, reinforcing the need for specific control measures at this point. The analysis of operational variables revealed that high slaughter speed and greater carcass weight were associated with higher *Salmonella* counts, particularly during critical stages such as evisceration.

There was a predominance of the serovar *Salmonella* Heidelberg, detected at all sampling points and throughout the weeks of analysis. The high genetic similarity among isolates obtained from different sampling points and collection dates demonstrated the occurrence of closely related profiles during the study period. Thus, this study reinforces that the control of *Salmonella* spp. in broiler slaughter requires an integrated approach that considers not only the presence of the pathogen, but also its microbial load and the operational factors involved in the process. The use of quantitative and genotypic methods contributed to a better understanding of contamination dynamics and may support sanitary control strategies in the poultry industry.
